# The Neuroscience of Spatial Navigation and the Relationship to Artificial Intelligence

**DOI:** 10.3389/fncom.2020.00063

**Published:** 2020-07-28

**Authors:** Edgar Bermudez-Contreras, Benjamin J. Clark, Aaron Wilber

**Affiliations:** ^1^Canadian Centre for Behavioural Neuroscience, University of Lethbridge, Lethbridge, AB, Canada; ^2^Department of Psychology, University of New Mexico, Albuquerque, NM, United States; ^3^Department of Psychology, Program in Neuroscience, Florida State University, Tallahassee, FL, United States

**Keywords:** neuroscience, artificial intelligence, spatial navigation, deep learning, reinforcement learning, memory, learning

## Abstract

Recent advances in artificial intelligence (AI) and neuroscience are impressive. In AI, this includes the development of computer programs that can beat a grandmaster at GO or outperform human radiologists at cancer detection. A great deal of these technological developments are directly related to progress in artificial neural networks—initially inspired by our knowledge about how the brain carries out computation. In parallel, neuroscience has also experienced significant advances in understanding the brain. For example, in the field of spatial navigation, knowledge about the mechanisms and brain regions involved in neural computations of cognitive maps—an internal representation of space—recently received the Nobel Prize in medicine. Much of the recent progress in neuroscience has partly been due to the development of technology used to record from very large populations of neurons in multiple regions of the brain with exquisite temporal and spatial resolution in behaving animals. With the advent of the vast quantities of data that these techniques allow us to collect there has been an increased interest in the intersection between AI and neuroscience, many of these intersections involve using AI as a novel tool to explore and analyze these large data sets. However, given the common initial motivation point—to understand the brain—these disciplines could be more strongly linked. Currently much of this potential synergy is not being realized. We propose that spatial navigation is an excellent area in which these two disciplines can converge to help advance what we know about the brain. In this review, we first summarize progress in the neuroscience of spatial navigation and reinforcement learning. We then turn our attention to discuss how spatial navigation has been modeled using descriptive, mechanistic, and normative approaches and the use of AI in such models. Next, we discuss how AI can advance neuroscience, how neuroscience can advance AI, and the limitations of these approaches. We finally conclude by highlighting promising lines of research in which spatial navigation can be the point of intersection between neuroscience and AI and how this can contribute to the advancement of the understanding of intelligent behavior.

## Introduction

Artificial General Intelligence (AGI), understood as the capability to produce intelligent behavior at the level of humans, has been a matter of debate. Even at the operational level, a definition that we can use to classify the behavior of any agent as intelligent or not, lacks consensus. Some define intelligence as the whole coordination of brain, body, and environment (Pfeifer and Scheier, 1999). Others also require the existence of a task to define intelligence (Almássy et al., 1998). From this perspective, in the absence of the elements of this definition of intelligence, adaptive intelligent behavior does not exist (Chiel and Beer, 1997). Another perspective is that intelligence can exist without actuators and even without an environment. The common denominator across perspectives and fields is that intelligence requires a brain. If we accept that premise, in order to understand intelligence, natural, or artificial, we must study the brain. The goal of neuroscience is precisely that—to understand how the brain works. If we assume that intelligent behavior can be understood by studying how it emerges, it is reasonable to attempt to learn from a working example: biological brains.

Historically, Artificial Intelligence (AI) researchers followed this approach. In fact, many of the initial ideas of numerous state-of-the-art algorithms in AI were derived from psychology and neuroscience (Hassabis et al., 2017). For example, Artificial Neural Networks (ANNs) were initially proposed in the 1940's, inspired by the organization and learning mechanisms observed in the brain (McCulloch and Pitts, 1943; Hebb, 1949). Up until a few years ago, ANNs were mainly used by academics. However, the recent success of Deep Learning (DL) in real-world problems has led to growing interest in industry that has fueled an unprecedented growth of artificial neural networks research (Sinz et al., 2019). In particular, DL and Reinforcement Learning (RL) have received a great deal of attention, not only from the scientific community but also from the general public due to the diverse sectors on which they are being applied such as health care, finance, and technology. DL is an area of machine learning in which ANNs with multiple layers are used to extract high-level features from their inputs (i.e., “deep”). RL on the other hand, is a methodology inspired by conditioning experiments in psychology in which agents learn to maximize rewards and minimize punishment by interacting in their environments (Sutton and Barto, 2018).

Despite the undeniable success of machine learning applications to diverse problems and progress in this field with regard to improving the algorithms and technology to train ANNs—which sometimes has lead to misleading remarks in the media about the real state of the field – there are criticisms about the real advancement in the AI field. One of the main criticisms is the lack of generalization and the amount of training examples that DL algorithms require for learning to solve even simple and structured tasks. In contrast, biological systems can learn complex tasks quickly and extract semantic knowledge from a relatively small number of instances. From this point of view, AI can greatly benefit from applying general principles that real brains employ to solve complex tasks.

In parallel to the advances in AI, the field of neuroscience has experienced tremendous progress in recent years due to the technological advances that allow high density recordings of brain activity with unprecedented spatiotemporal resolution from multiple parts of the brain simultaneously (Steinmetz et al., 2018). Some of these recent advances in the neuroscience of spatial navigation led to the Nobel Prize in Medicine being awarded to John O'Keefe and Edvard and May-Brit Moser (Colgin, 2019). Despite this notable progress in neuroscience, there is a concern that with big data, the field needs to improve the analytical tools to help us make sense of the complex and vast quantities of data that we are now capable of recording (Jonas and Kording, 2017; Vogt, 2018; Vu et al., 2018). Besides analytical tools to make sense of the data, there is a need for tools that help generate new hypotheses based on these richer and larger datasets.

With progress in both Neuroscience and AI, there is a recent renewed interest to conduct research bridging these two fields so that they may benefit from each other (Hassabis et al., 2017; Jonas and Kording, 2017; Richards et al., 2019). One obvious and already successful interaction between AI and Neuroscience is to use machine learning (ML), an area of AI that applies computer science and statistical techniques in data analysis, to study complex and large datasets in Neuroscience (Vogt, 2018; Vu et al., 2018). For example, ML methods are used to analyze motion characteristics of behaving animals to predict cognitive function (Ryait et al., 2019), and have been used in studies of sensory processing to determine optimal stimuli for representations in primary visual cortex (Walker et al., 2019). Besides using ML as an analytical tool, there are attempts to go further and use artificial neural networks as a model to understand brain function (Musall et al., 2019; Richards et al., 2019).

A potential convergent point in which neuroscience and AI could be combined to learn more about the brain, and advance both fields, is spatial navigation. From a neuroscience perspective, being able to actively explore the world might have been one of the key factors that provided organisms evolutionary advantages that triggered the development of cognitive process such as prediction, attention, learning and memory (Swanson, 2003). For example, it has been hypothesized that the memory processing mechanisms involving the entorhinal cortex and the hippocampus evolved from the mechanisms that compute the relationships of spatial landmarks and the tracking of movements of the body in the world (Buzsáki and Moser, 2013). Even more, these computations enabling neural representations of the animal location carried out in the parietal and entorhinal cortices have been proposed as a general mechanism implemented across the neocortex to represent spatial relationships between objects and as a general mechanism for many conceptual “spaces” (Constantinescu et al., 2016; Behrens et al., 2018; Hawkins et al., 2019). Therefore, by understanding how spatial navigation is carried out in biological systems, we can learn about the underlying cognitive processes that are also important components of intelligent behavior which may further advance AI (Bellmund et al., 2018).

In this paper we propose spatial navigation as a common ground for neuroscience and AI to converge and exchange ideas and expand our knowledge of the brain and, ultimately, complex intelligent behavior. In the following sections we first go into detail about why spatial navigation could be used to learn about the brain and advance AI. Then we review the neurobiology of the rodent spatial navigation system, highlighting the structures that form the main concepts of what we know about space representations in the brain: head-direction, place, grid, and border cells. After, we review the models used to study these structures and the processes involved in spatial navigation. Finally, we highlight the limitations of the proposed approach and conclude by providing future directions in which a closer interaction between the fields could improve our understanding of the brain and ultimately of intelligent behavior.

## The Neuroscience of Spatial Navigation

In order to describe how neuroscience and AI are well-suited to benefit from one another, we begin by summarizing what is known about the neuroscience of spatial navigation. Here we briefly describe some of the key findings about the neural correlates of spatial navigation and the computational bases of these neural substrates. In addition, we also provide a summary of the neuroscience of reinforcement learning which is a key ingredient in the development of newer AI approaches to understand how spatial navigation tasks might be solved by biological systems. This section aims to denote the limits of what is known about the neurobiology of spatial navigation but also provides important directions and constrains that models should take into consideration.

**Figure 1:**
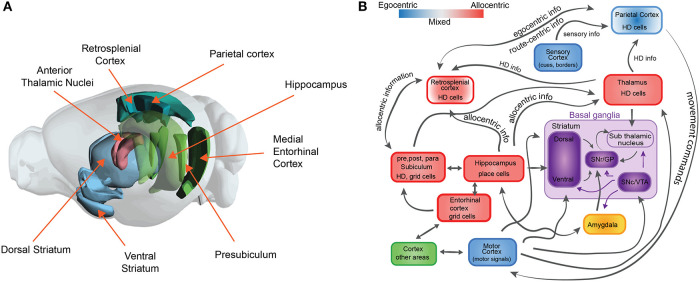
The neuroscience of spatial navigation. **(A)** Key brain structures involved in rodent spatial navigation. Diagram adapted from the Allen Brain Atlas Explorer. **(B)** Schematic illustrating the general pattern of anatomical connectivity and the functional shift in frames of reference encoded by the brain regions that comprise the neural circuitry of spatial navigation. Hippocampus (HPC) and parahippocampal regions (entorhinal cortex, postsubiculum, and parasubiculum) encode an animal's position in space predominantly in allocentric or map-like coordinates. The blue and red boxes represent a spectrum denoting the relative density of egocentric (viewer-dependent, self-centered, or action centered frame of reference) vs. allocentric (map-like) encoding for each region. Retrosplenial cortex (RSC). Parietal cortex (PC) and anterior thalamic nucleus (ATN) are anatomically and functionally well-positioned to interface between egocentric and allocentric frames of reference within a larger navigational network. Purple boxes represent brain structures involved in value-based signals for conditional learning and spatial navigation (The basal ganglia circuit sub-diagram was inspired from Chersi and Burgess, 2015, used with permission).

First, it is important to note that accurate navigation involves several different strategies to reach a goal location: one can follow a sensory cue that marks a goal location, one can follow a determined sequence of actions (a route), or one can determine which way to proceed by following an internal representation of space (map). Depending on the strategy, multiple cognitive processes (or combinations) are required and therefore involve coordination across several brain regions (see Figure 1). In addition, spatial navigation involves several cognitive processes that are crucial for a broad range of intelligent behavior. For example, spatial navigation is strongly linked to memory and learning, planning, attention, and decision making, among others (O'Keefe and Nadel, 1978; Gallistel, 1990; McNaughton et al., 1991; Skaggs and McNaughton, 1996).

Second, spatial navigation has been proposed to follow two different complementary learning strategies that reflect the processes that are computed in the hippocampus and the striatum (Chersi and Burgess, 2015). Briefly, when the striatum is involved, local and incremental reinforcement learning rules facilitate subsequent learning of spatial navigation tasks based on egocentric information (Figure 1A). In this type of navigation, loops between the cortex and the basal ganglia are proposed to support stimulus-response associations and procedural memory, which are linked to route or cue-based navigation. In contrast, when the hippocampus is involved, faster one-shot associative learning rules are applied to solve spatial navigation. Recent studies in humans link these mechanisms for decision making, in which model-free choice guides route-based navigation and model-based choice directs map-based navigation (Anggraini et al., 2018).

### Spatial Navigation and the Neural Basis of the Cognitive Map

The theory of the “cognitive map” proposes that the brain creates a representation (or model) of the environmental space that is used to navigate (Tolman, 1948; O'Keefe and Nadel, 1978; Gallistel, 1990). Experimental support for this theory is derived from studies that require rodents (and humans) to solve navigational tasks where the goal location is not visible from an animals current location (Knierim and Hamilton, 2011). Navigation to precise “hidden” locations, or place navigation, can be performed by referencing distant landmarks (or allocentric frame of reference), or by referencing one's body orientation in relation to cues and executing a sequence of actions to the goal (an egocentric frame of reference). Some theoretical work suggests that allocentric and egocentric frames of reference can operate sequentially such that information is decoded to determine a subject's egocentric orientation in the environment and vice versa (McNaughton et al., 1995; Byrne and Becker, 2007; Burgess, 2008; Clark et al., 2018). For example, an allocentric to egocentric transformation may allow a subject to select an action (turn left) at a specific intersection (a particular allocentric location and orientation) in a city. In addition, animals can localize their position and produce trajectories to goal locations by using self-motion cues, e.g., vestibular, proprioceptive, optic flow often referred to as path integration or dead reckoning (Gallistel, 1990; McNaughton et al., 1991; Whishaw et al., 2001).

The neurobiological basis of map-like spatial representations is thought to involve a network of spatially selective neurons in the mammalian nervous system. These include populations of cells that code for spatial location such as place cells (O'Keefe and Nadel, 1978), grid cells (Hafting et al., 2005; Bonnevie et al., 2013), border cells (Solstad et al., 2008), landmark or object vector cells (Deshmukh and Knierim, 2013; Wilber et al., 2014; Høydal et al., 2019), cells that code for head direction (Taube et al., 1990), cells that code for an animals egocentric orientation with respect to environmental features (Wilber et al., 2014; Hinman et al., 2019; LaChance et al., 2019; Alexander et al., 2020), position along a route (Nitz, 2006), and angular and linear locomotor speed (McNaughton et al., 1994; Sharp et al., 2001; Wilber et al., 2014, 2017; Kropff et al., 2015; Munn et al., 2020). These spatial cell types have been identified in a neural circuit that includes the hippocampal formation and several limbic-thalamic and limbic-cortical regions (see Figures 1A,B). While hippocampal circuitry has been linked with allocentric spatial processing, subcortical regions such as a basal ganglia-cortical circuit are thought to contribute to some forms of egocentric action-based navigation. The latter circuit has also been associated with stimulus-response learning, procedural memory and reward prediction. We briefly describe these spatial cell types in greater detail below to provide relevant biological restrictions that can be used in the development of models to study spatial navigation that can inform neuroscience.

#### Place Cells and Grid Cells

**Figure 2:**
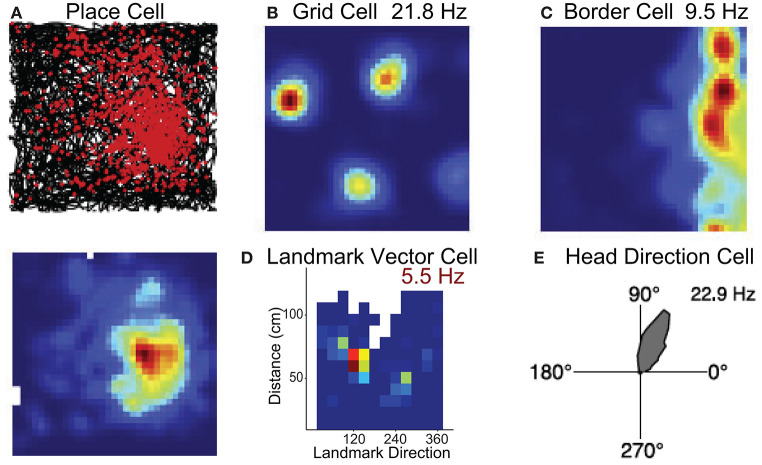
Neural substrates of spatial navigation. Five examples of single units that exemplify the encoding present in several regions that encompass the brain network critical for spatial navigation. **(A)** Example place cell recorded in hippocampus, top row is a spike/path plot, red dots represent the locations of action potentials and black lines the path of the animal. **(B,C)** Example grid cell and border cell recorded in parahippocampal cortex. Colormaps are standard evenly spaced colormaps and the peak firing rate is indicated. **(D)** Colormap for a cell in hippocampus that encodes the direction and distance of an environmental landmark. Data are from Wilber et al. (2014). **(E)** Polar plot showing firing rate by HD for an HD cell. Firing rate (Hz) is represented in upper right corner for each example cell. Data in **(A–C,E)** are from Harvey et al. (2019).

The hippocampus contains neurons that discharge in specific environmental locations (Figure 2A; O'Keefe and Dostrovsky, 1971) such that populations of these cells encode the present position much like a GPS (O'Keefe and Nadel, 1978). Grid cells have been identified in parahippocampal cortex (medial entorhinal cortex, presubiculum, parasubiculum) and differ from place cells in that they fire in multiple locations forming a hexagonal grid pattern (Figure 2B; Hafting et al., 2005). The location by which place/grid cells form their firing fields are modulated by self-motion stimuli or path integration (McNaughton et al., 2006), and are also modulated by landmarks such as local or distant environmental cues or its overall shape (O'Keefe and Burgess, 1996; Yoder et al., 2011; Krupic et al., 2015).

#### Border, Landmark or Object Vector Cells

While place and grid cells can be modulated by the shape of the environment, recent work has shown that the parahippocampal cortex (medial entorhinal cortex, presubiculum, parasubiculum) also contains neurons that respond specifically to boundary stimuli (Figure 2C; Solstad et al., 2008; Boccara et al., 2010). In the subiculum, these “border” cells can also discharge at specific distances relative to a boundary (Lever et al., 2009). In addition, cells have been identified in both the hippocampus and medial entorhinal cortex that discharge at a specific direction and distance in relation to a cue (Figure 2D). These place responses have been described as landmark or object vector cell activity (McNaughton et al., 1995; Deshmukh and Knierim, 2013; Wilber et al., 2014; Høydal et al., 2019).

#### Head Direction Cells

Several interconnected limbic and parahippocampal regions contain populations of neurons termed head direction (HD) cells (Cullen and Taube, 2017; Peyrache et al., 2019; Angelaki and Laurens, 2020; Munn and Giocomo, 2020). HD cells are neurons that fire maximally when an animal points its head in a specific direction (Figure 2E) and a small population of HD cells can accurately track the animals HD (Peyrache et al., 2015; Xu Z. et al., 2019). Research has identified this signal in the anterior thalamic nuclei, retrosplenial, parietal, and parahippocampal (entorhinal, postsubiculum, and parasubiculum) cortices (Taube, 2007). Similar to location specific firing in the hippocampus and parahippocampal cortex (place, grid, border, and object vector cells), the preferred direction of HD cells can be controlled by self-motion cues (angular path integration) and environmental cues (reviewed in Taube, 2007).

#### Egocentric, Action, and Route Modulated Cells

Recent work has established that a circuit including the parietal and retrosplenial cortex and neighboring hippocampal and subcortical regions play a central role in processing an egocentric coordinate system (Clark et al., 2018; Wang et al., 2018, 2020; Hinman et al., 2019; LaChance et al., 2019). For instance, neural populations in the parietal and retrosplenial cortex fire in response to an animal's egocentric actions or posture (McNaughton et al., 1994; Whitlock et al., 2012; Wilber et al., 2017; Mimica et al., 2018), egocentric orientation relative to a landmark or environmental boundary (Wilber et al., 2014; Alexander et al., 2020), and location along a complex route (Nitz, 2006). The parietal cortex has also been linked to allocentric information processing with some parietal neurons exhibiting allocentric HD correlates, and others modulated by the conjunction of egocentric and HD correlates (Chen et al., 1994; Wilber et al., 2014). Importantly, these conjunctive cell populations and other cells encoding primarily in action centered coordinates anticipate upcoming actions, for example, anticipating a left or right turn (Whitlock et al., 2012; Wilber et al., 2014). In addition, recent work has shown that retrosplenial neurons exhibit HD, position, and spike in the relation to the animals distance between path segments, as well as a conjunctive combination of these firing characteristics (Alexander and Nitz, 2015; Mao et al., 2017, 2018). Thus, the parietal and retrosplenial cortex may be part of a circuit that interfaces between allocentric and egocentric frames of reference (Pennartz et al., 2011; Stoianov et al., 2018). Therefore, these computations performed in the parietal and retrosplenial cortex might be crucial for understanding how transformations between self-centered experiences is related to map-like representations of space.

In summary, the mammalian nervous system encodes a map-like representation of space. This map-like representation is built from cells that code for: spatial location such as place, head direction, the position in the environment with repeating geometric firing patterns—grid cells, borders, the direction and distance of landmarks or objects, an animals egocentric orientation with respect to environmental features, position along a route, and specific combinations of angular and linear locomotor speed. The cells that encode space in allocentric or map-like coordinates are generally found in the hippocampal formation and several limbic-thalamic and limbic-cortical regions. While the cells that encode egocentric or body-centered coordinates are generally found in subcortical regions such as a basal ganglia and posterior cortical regions such as the parietal cortex. The computations carried out by the cells and circuits involved in spatial navigation presented here are thought to be similar to the substrates for cognitive processes such as memory, semantic knowledge extraction, prediction, and decision making which in turn are building blocks of intelligent behavior.

### Spatial Navigation and Reinforcement Learning

Another important cognitive process strongly linked to spatial navigation and important in intelligent behavior is learning. The brain structures that are involved in spatial navigation and memory formation are also involved in learning (Bellmund et al., 2018). During spatial navigation, learning can occur first as a trial-and-error process that links memory and reward or punishment signals. However, later with experience, a cognitive map is formed and can be used to infer useful spatial information (Buzsáki and Moser, 2013). This adaptive process exploits previous experience to improve the outcome of future choices using different strategies that are implemented in different areas of the brain, including the hippocampus (Johnson and Venditto, 2015). Covering all aspects of learning underlying spatial navigation is beyond the scope of this review where we will focus an aspects that is amenable to AI approaches, RL. We briefly summarize the area of reinforcement learning and the brain structures that are involved in the process of sequential decision making that are crucial to navigate.

In RL there are two main approaches to implement learning, model-free and model-based (and also hybrid approaches). In model-free learning, there is no representation of the world. Instead, learning happens based on more immediate sensory-actions associations. In contrast, in model-based learning there is an internal representation of the environment (Lee et al., 2012). These two approaches nicely overlap with the egocentric and allocentric frames of reference for spatial navigation and have been proposed to work together (Khamassi and Humphries, 2012). Model-free learning is proposed to be implemented by cortical-basal ganglia loops that participate in sensory-actions associations used to update the value function (Figure 1B). In spatial navigation, analogous computations are thought to be egocentric (or route-based) in which no cognitive map is used to reach a goal location. Instead, in model-based learning the animal (agent) uses an internal representation of the environment to update the value function. This representation corresponds to the cognitive map in spatial navigation in which the position of the animal in the environment is updated. In this case, these processes are thought to be implemented by the interaction between the hippocampus and the ventral striatum (Pennartz et al., 2011). In AI and neuroscience, research in RL and rodent spatial navigation has already proved to be a successful approach to understand how these two concepts are closely related and the interaction between these fields can help to inform one another. For example (Stoianov et al., 2018), demonstrated how a RL model can replicate results in rodent experiments in which contextual cues are manipulated to explore the behavioral and brain constrains in goal directed navigation tasks.

Although more research is needed to clarify the details about the neuroscience of the interaction between the navigation and learning systems, there is increasing progress in this area. For example, it is known that the basis of route-based navigation involves brain structures which encode sensory-action associations such as the striatum. In particular, the sensory information reaches the dorsal striatum from the corresponding cortical areas. The dorsal striatum sends projections to the substantia nigra reticulata, which in turn receives inputs from the substantia niagra compacta and the ventral tegmental area (VTA) (Figure 1B). The reward signals are thought to originate in the VTA. Therefore, it has been hypothesized that, since the ventral striatum receives direct input from the SNc/VTA and the hippocampus, the associations between place and reward signals are performed in the latter structure (Chersi and Burgess, 2015).

In summary, different structures interact in spatial navigation and learning depending on the strategy used. Although more research in this area is needed, this section provides a brief review of the neuroscience underlying this ability within the context of RL. This knowledge can inform and guide some of the parameters used in artificial agents solving spatial navigation tasks. This informed development of AI systems will in turn provide opportunities to validate the result against rodent experiments and therefore, to generate hypothesis that can inform neuroscience.

## Models for Spatial Navigation and Their Contribution to the Understanding of the Brain

Major advances in our understanding of how the brain is involved in spatial navigation has been achieved in part, due to modeling work. Depending on what the goal of the model is, it can be classified as descriptive, mechanistic, or normative (Dayan and Abbott, 2001). Descriptive models of spatial navigation have the goal of characterizing what the system does, usually reproducing experimental data (Sutherland and Hamilton, 2004). Mechanistic models of the spatial navigation system provide an explanation of how spatial navigation is solved using processes and mechanisms. In this modeling approach, explicit implementations and assumptions are derived from observations and hypotheses from experimental work. Here we will use the term “hypothesis driven models” to encapsulate both descriptive and mechanistic models. Finally, in normative models (or end-to-end models in AI) of spatial navigation the goal is to understand why the brain might solve spatial navigation in a particular way, so the assumptions are less explicit. In this section, we describe the modeling work that encompasses all these approaches that have been developed to understand how animals navigate in space. In addition, we describe modeling work on reinforcement learning which has been important for the development of end-to-end AI approaches that tackle spatial navigation tasks.

### Hypothesis-Driven Models of Head-Direction, Place and Grid Cells for Spatial Navigation

When navigating using path integration, it is necessary for the brain to encode the spatial location and update this information with the direction and the speed of motion. Hippocampal place cells are thought to provide this critical information. The modeling complexity of the activity of place cells largely varies depending on the goal of the study. For example, place cells have been modeled as an arrangement of radial basis functions (e.g., Gaussian) each centered at its corresponding place field and collectively the set of place fields cover the environment (Bonnevie et al., 2013; Cazin et al., 2020). In these cases, the position of the agent has been modeled as the actual position in the environment (Arleo and Gerstner, 2000). In other models for which the goal is to study the spatial representations, the current position and distance from the centers of the place field is derived from sensory and idiothetic information (Banino et al., 2018; Cueva and Wei, 2018).

**Figure 3:**
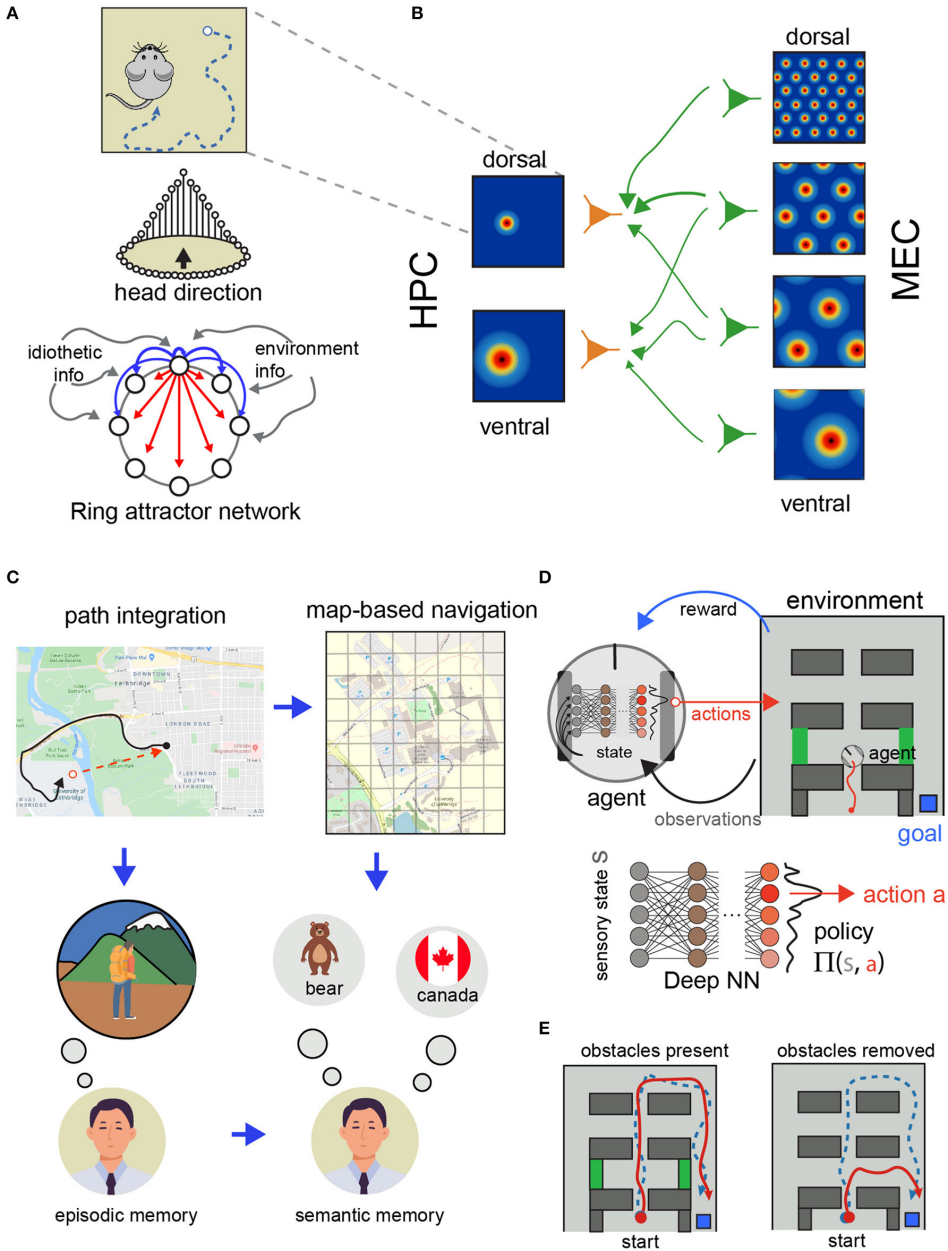
Models of spatial navigation and their relationship with reinforcement learning. **(A)** Path integration requires keeping track of the turns and distances traveled as the animal explores the environment (top). Ring attractor network model of head-direction (bottom). The idiothetic and environmental information update and rectify the spatial representations in the model. Inspired by Schultheiss and Redish (2015), used with permission. **(B)** Grid cells in the medial entorhinal cortex (MEC) at different scales (top) and place cells in the hippocampus (HPC) with different scales (bottom). Having access to different scales allows the system to represent space at different resolutions. Adapted from Solstad et al. (2006), used with permission. **(C)** Relationship between episodic and semantic memories and path integration and model-based navigation. Inspired from Buzsáki and Moser (2013). **(D)** Schematic representation of the deep RL approach for spatial navigation. A deep NN is used to estimate the best action to execute to maximize future rewards. **(E)** Example trajectories of two agents trained using place and head direction cells in Banino et al. (2018). Some of the agents developed grid-cell like representations (red) and others only place and head direction cells-like representations (blue). During learning both agents were able to reach the goal (top). During testing, when obstacles where removed, only the agents using grid-like representations used shorter routes (bottom). Diagram adapted from Banino et al. (2018), used with permission.

In turn, HD cells have been modeled as a recurrent network that maintains a representation of the current orientation and is modulated by self-motion signals (Figures 2E, 3A). The architecture of this network has been proposed as a ring attractor network (Skaggs et al., 1994; McNaughton et al., 2006; Clark and Taube, 2012; Knierim and Zhang, 2012). Briefly, these models involve HD cells conceptually arranged in an anatomically connected ring with each HD cell occupying a position corresponding to their preferred firing direction. Thus, adjacent HD cells on the “ring” share similar, but slightly offset, preferred firing directions (though not necessarily physically adjacent positions in the brain). The anatomical ring is organized such that functionally adjacent HD cells share strong excitatory connections while cells that occupy different directions (e.g., are 180 degrees apart) share weaker excitatory connectivity (or are inhibited). A consequence of this framework is a sustained hill of excitation centered on the animal's current HD. In most models, external inputs from environmental cues and angular head velocity derived from idiothetic self-motion cues (angular path integration) move the activity hill around the ring (Taube, 2007). Thus, the activity hill is organized to move corresponding to the animal's current HD.

Besides these models of head orientation, there is an extensive body of modeling work to understand how place is represented in the brain. Modeling work has followed two approaches to study the organization of grid cells (Figure 3B). One in which a recurrent network is optimized with hand-chosen parameters to reproduce the hexagonal pattern of activation observed in electrophysiological recordings (McNaughton et al., 2006; Giocomo et al., 2011; Knierim and Zhang, 2012; Navratilova et al., 2012). This approach has been shown to work in simulated conditions (Samu et al., 2009). In these simulations allocentric information derived from a model of grid cells during path integration can correct the accumulated error generated by a noisy representation of speed and direction. In a more recent model (Bush et al., 2015) modeled grid cells to support vector navigation and provide provide a framework for testing experimental predictions. As the neuroscience of spatial navigation uncovers more about how space is represented and manipulated in the brain, models reflect this progress as well. For example, with more studies about grid cells, models that aim to understand how place cells and grid cells interact have been very important to understand the restrictions in the circuitry between the enthorinal cortex and the hippocampus (Solstad et al., 2006).

### Hypothesis Driven Models of Spatial Reference Frame Coordination for Navigation

Spatial navigation systems, in mammals at least, are highly robust and adaptable to different levels of sensory information and environmental conditions. For example, mammals are capable of navigating in darkness using internal representations of space or using sensory cues and are capable of rapidly updating these representations when distant cues and landmarks are available (Rosenzweig et al., 2003). These complex navigational strategies rely on the ability to change the frames of reference to use spatial information (Figure 3C). In this section we review the modeling work developed to understand how the brain transforms spatial information between different frames of reference. This is important not only to understand the brain but also because these transformations can be important to extract knowledge by deriving semantic knowledge form episodic memories (Figure 3D; Buzsáki and Moser, 2013; Wang et al., 2020).

#### Allocentric-Egocentric Reference Frame Transformation

Computational descriptive models propose that cell populations within the anterior thalamic nuclei, parietal cortex, and retrosplenial cortex operate as a network that transforms spatial information from an egocentric (e.g., body centered) to allocentric (i.e., map-like) frame of reference and vice versa (reviewed in Clark et al., 2018). For example, the view of a landmark, which is body-centered in nature, can be transformed into an allocentric, map-like, frame of reference. The details of these reference frame transformations vary slightly between models but are similar regarding the neurobiological subcomponents. For example, in one variant of this framework, McNaughton et al. (1995) model an egocentric-to-allocentric transformation using a linear mapping across a three-layered neural circuit. The initial computations involve a layer composed of two neural populations: an allocentric HD cell signal, which is generated within a subcortical circuit including anterior thalamic-to-cortical projections, and an egocentric cell signal by parietal cortex neurons which are modulated by the animals' egocentric heading relative to a landmark. These two cell types converge post-synaptically on a second layer of parietal cortex cells that encode the conjunction of HD and egocentric signals. The biological validity of these conjunctive cells is supported by recent work (Wilber et al., 2014). Conjunctive cells are then associated with a third layer encoding the bearing of a landmark (allocentric direction), which when combined with information regarding the relative distance from the landmark, produces a signal reflecting a vector relative to the landmark. In sum, this model demonstrates how sensory information can be used to support spatial localization by transforming egocentric information into a location in the environment. In other words, transform body centered encoding of a landmark into map-like landmark representations (e.g., a cell that fires in a specific map-like location relative to a landmark independent of which direction the animal is facing; Figure 2D). Recent studies have identified neural correlates resembling exactly this model output, or landmark vector cell responses, within the hippocampus and entorhinal cortex (Deshmukh and Knierim, 2013; Wilber et al., 2014; Høydal et al., 2019). In addition, the opposite transformation in which allocentric location is decoded to determine egocentric orientation could be performed using a similar 3-layer network. For instance, if a subject is required to select the appropriate action (turn left) for a specific intersection in a city (a specific place and direction) given the current goal (e.g., to go to the bank vs. coffee).

There are mechanistic modeling studies in which spatial representations found in the mammalian navigation system are used to study how different frames of reference can be used to navigate when different sensory information is available. For example (Byrne and Becker, 2007), implemented a model that shows how egocentric and allocentric frames of references can be built and how transformation from one to another can be carried out. Moreover, in a related study, it is shown how these transformations relate to short and long-term memory (Krichmar and Edelman, 2005). More recently (Oess et al., 2017), showed how the hippocampus, the parietal cortex and retrosplenial cortices could interact to solve spatial navigation tasks using an egocentric, an allocentric or route-centric frames of references. These simulations can help to understand how the transformation of egocentric and allocentric frames of reference can be employed by the brain when using different navigation strategies. Another example in which modeling aspects of the rodent spatial navigation system has helped to understand the integration of self-motion and visual information to represent the localization in space is by using an attractor-based network model (Campbell et al., 2018). In this work they use this model to understand how optic-flow, locomotion and landmark cues produce activity patterns in the medial entorhinal cortex to represent spatial position during navigational tasks.

### An ANN Model Approach to Solve Spatial Navigation

In a traditional approach to modeling brain function, such as spatial navigation, the model parameters are specified by the experimenter and optimized to reproduce experimental data. More recently, with the advancement in ANNs, there are more AI end-to-end (normative) approaches to model spatial navigation in which the parameters that determine the representations and how they are exploited are not specified explicitly. Instead, ANNs are trained to solve spatial navigation tasks and the representations and parameters employed by the network are restricted to match biological constraints. In this section we recapitulate modeling work that has the goal of advancing the understanding of the rodent spatial navigation system using both AI and neuroscience approaches.

The spatial representations that ANNs use to solve spatial navigation resemble different properties reported in rodent experiments. For example Kanitscheider and Fiete (2017), trained a RNN to perform a series of spatial navigation tasks that require self-localization and mapping, a very well-studied and useful property in robotic navigation. In this work, the authors show that the representations that are exploited by the trained network resemble characteristics of the biological spatial navigation system such as place cells that remap between environments (thought to represent the neural substrate of unique cognitive maps for different locations). Similarly Cueva and Wei (2018), showed how an agent using a recurrent neural network (RNN) can solve a spatial navigation task to study the spatial representations used by such network. In the trained RNN, they found a grid-cell like representation of space in which a hexagonal periodic pattern of activity was used to keep track of the location of the agent in the environment. The spatial representation exploited by this network did not combine the sensory raw input and motion signals as in other models (Samu et al., 2009). Instead, this representation emerged because of the integration of the speed and direction signals given to the network and the metabolic restriction implemented in the cost function used to train the network. One limitation of these RNN approaches is that the learning mechanism used to train the agent to solve the task were not biologically plausible.

Recently, there are studies in which applying biologically relevant restrictions to the ANNs led to understanding how these processes occur in the brain. For example Whittington et al. (2018), propose a model inspired by the hippocampal-entorhinal cortex system in which grid and place cell like representations emerge when an ANNs is trained to solve navigation in a 2D environment. These representations constructed by the ANN using the end-to-end approach, generalizes from sensory exposures from different environments. Moreover Sorscher et al. (2019), analyzed the conditions in which grid cell-like representations emerge from models optimized for spatial navigation. In this work, the authors were able to ascertain which constraints favor the hexagonal activation pattern of grid cell like representation emerged in three different network architectures.

In summary, this end-to-end approach in which ANNs are used to model brains in embodied agents that learn to navigate in space using relevant biological restrictions provides a promising tool to study the representations of space that might resemble those used in nature and further our understanding of how such spatial representations may “emerge.”

### Reinforcement Learning and Spatial Navigation

An important related element in the mammalian spatial navigation system is learning. One way to model how embodied agents learn to navigate is using RL. In this section we summarize how RL modeling has been successfully integrated in AI approaches to understand spatial navigation.

Recently, DNNs have been combined with RL (termed as Deep RL) in spatial navigation tasks. In this framework, spatial representations are learned by interacting with the environment instead of provided by the experimenter. In this version of RL, deep NN are used to approximate value and transition functions using environmental (sensory) information (instead of a look up table or another function approximation method) (Botvinick et al., 2019). Due to considering multiple layers (i.e., deep), deep RL leverages this organization to learn spatial representations that generalize well and can be transferred to different tasks (Mirowski et al., 2017; Banino et al., 2018; Botvinick et al., 2019). For instance Banino et al. (2018), used deep learning in simulated agents to study how space representations can be used to facilitate flexible navigation strategies that closely resemble experimental data from rodents. In this work, the authors trained the deep network to perform path integration using trajectories from real rodents. One of the intermediate representations that the simulated agents used to keep track its location when doing path integration was grid cell-like activity patterns. Moreover, the authors investigated whether this representation could be exploited to perform goal directed navigation. This time, the authors included a RL module which learned to associate values to specific locations in the environment. Actions that brought the agent closer to the goal were associated with higher value. Using this strategy, these agents solved the goal directed navigation tasks using shortcuts when possible, compared to agents only using place and head direction cells to navigate (Figure 3E). This type of work in which similar representations to the ones found in real brains are used to solve navigation tasks is important because they provide opportunities to learn more about how similar processes might happen in the brain.

Besides using ANNs and RL to solve spatial navigation tasks, important concepts, and mechanisms found in neuroscience experiments have been used to improve algorithms in AI. For example, an important mechanism that links what we know about learning and memory and spatial navigation are the theories about how memories are consolidated in the brain. In one theory about memory, hippocampal replay plays a crucial role in forming an index or memory trace that binds together experience components in the neocortex for long-term storage and knowledge extraction during sleep (Frankland and Bontempi, 2005). There are numerous examples of experimental evidence that replays occurs in multiple areas of the brain (Skaggs and McNaughton, 1996; Kudrimoti et al., 1999; Euston et al., 2007; Bermudez Contreras et al., 2013; Wilber et al., 2017). This replay mechanism has been proposed to reduce the number of “iterations” required to explore an environment in RL (Cazé et al., 2018; Momennejad et al., 2018; Cazin et al., 2019).

Finally, despite the contribution of more abstract models of spatial navigation, there are models in which AI takes a more ethological and embodied approach to study spatial navigation. From this perspective, cognition is not only a product of isolated computations occurring in the brain but instead emerge from the interaction between the body and the environment (Noe and O'Regan, 2001; Thelen and Smith, 2007; Bonner and Epstein, 2017). Therefore, from this perspective, instead of solely looking for the responsible brain structures involved in spatial navigation and their neural codes, the study of the restrictions imposed by the environment and anatomy of the organism, might help to better understand how the internal representations are constructed and how they are manipulated to navigate in space (Krichmar and Edelman, 2005; Evans et al., 2016; Brette, 2019; Santoro et al., 2019).

We have summarized modeling work that has the goal of improving our understanding of the mechanisms involved in spatial navigation and how they are implemented by the brain. The range of such work varies from descriptive and mechanistic models in which the goal is to reproduce experimental data using explicit hypotheses about brain organization, to more recent approaches that rely less on explicit experimenter definitions and use ANNs as a model of the brain. In addition, we have summarized the neurobiology of RL and how RL has been implemented to solve spatial navigation tasks. Regardless of the level of abstraction and the questions they aim to answer, this work expands our knowledge of the brain by providing predictions, generating new hypotheses and demonstrating how the cognitive processes necessary for complex behavior might rise from spatial navigation.

## Discussion

Here we reviewed the neuroscience and modeling work of spatial navigation. We proposed that by understanding how spatial navigation is solved by the brain, we could provide useful insights to alleviate some current problems for AI. Conversely, a way to expand our current understanding of the neuroscience of spatial navigation is to use AI and machine learning techniques to aid in analyzing some large data sets. This has historically been demonstrated by testing theories of how the brain performs spatial navigation using descriptive and mechanistic models of the hippocampal formation. More recently, the development of end-to-end or normative approaches using ANNs to solve spatial navigation problems while following relevant biological restrictions can further our understanding of how and why representations might emerge and be manipulated by the brain. In this section we outline how considering spatial navigation as the intersection point between neuroscience and AI research can provide a valuable opportunity to advance both fields and we review the limitations of the approaches presented in this paper.

### Neuroscience Contributions to Advance AI

As previously mentioned, there are strong examples of contributions from neuroscience and psychology to the advancement of AI, such as the inspiration for connectionism and of ANNs (Rumelhart et al., 1988), the hierarchical organization of the mammalian visual processing in the cortex for the development of deep learning (Schmidhuber, 2014), the successful application of attentional mechanisms to active computer vision (Bermudez Contreras et al., 2008) or training ANNs (Graves, 2013; Sutskever et al., 2014), and the impressive development of RL systems that can beat world-class players at highly-complex games (Botvinick et al., 2019). Despite these contributions that have propelled the recent impressive progress and applications of DL and RL, there are important limitations in these areas which can benefit from the knowledge generated in neuroscience. First, a limitation of the current state of DL is that, despite being used in impressive applications, these networks suffer from poor generalization or fail to extract semantic knowledge from the large training data sets. For example, successful applications of classic DL are highly optimized non-linear classifier systems that require many training examples to fine tune a large number of parameters rather than systems that extract knowledge by building robust semantic understanding of the inputs. In contrast, the analogous biological networks show a great deal of generalization during learning. The way that the brain performs spatial navigation might provide valuable insights into how to solve this limitation in current AI methods. First, memory consolidation is hypothesized to use a mechanism in which relevant episodes (time and space dependent) are formed into memories from which semantic knowledge (context independent) is extracted. These mechanisms, possibly implemented in the same network, might be similarly employed for navigation in the formation of cognitive maps from repeated exposure to self-centered exploration episodes (Figure 3C; Lever et al., 2002; Buzsáki and Moser, 2013).

Another related limitation is the number of training examples that DL requires to learn. This limitation is contrasted with the biological counterparts in which learning happens very rapidly in most cases. This might be due to the fact that brains are not completely randomly connected at birth such that we have to learn everything from scratch. Instead, there are developmental processes that determine pre-wired networks and mechanisms that bootstrap innate behaviors (Zador, 2019). Thus, neuroscience may be able to inform AI so that models can combine learning with evolutionary and developmental approaches in which plasticity and circuit refinement build upon pre-wired brain networks. A similar limitation in RL arises in complex environments where agents require a large number of exposures to the environment in order to improve policies (which is the way that determines how the agent interact with its environment). A neuroscience-inspired mechanism to reduce the number of required exposures for learning that is also implemented by structures involved in spatial navigation, is to use previous experiences to select possible actions for new situations. This approach, known as learning to learn (episodic meta RL), has already been successfully applied to solve spatial navigation tasks (Chalmers et al., 2016, 2017; Duan et al., 2017; Finn et al., 2017; Botvinick et al., 2019). In addition, a well-studied process in neuroscience is memory consolidation in which the replay of previous experiences helps to extract semantic knowledge from episodic instances. There is also evidence that not only previous experiences but also unexplored possibilities are used to evaluate possible outcomes in navigation tasks in rodents (Dragoi and Tonegawa, 2011). A similar approach has been applied in ANNs to solve spatial navigation in simulated agents and robots (Cazin et al., 2019, 2020). Another important interaction between AI and Neuroscience in spatial navigation has been the idea that the hippocampus is not a spatial cognitive map but instead, a prediction map (Evans and Burgess, 2019). From this perspective, hippocampal activity encodes the animal's future locations which are restricted by the environment and their value (rewards) (Stachenfeld et al., 2017; Brunec and Momennejad, 2019).

An additional aspect in which neuroscience could provide ideas to advance AI and in particular ANNs is by incorporating what is known about brain architecture. At the moment, most of the deep learning approaches use a limited repertoire of what is known about how brain cells compute information. For example, the richness of neuronal types, network topologies or the additional dynamics provided by neuromodulators, the combination of local and long-range synaptic connections might improve the capabilities or reduce the limitations of AI approaches (Ullman, 2019). One important aspect of the representations derived from ANNs is their robustness. The generalization achieved by these systems can be enhanced using regularization techniques during training such as dropout (Srivastava et al., 2014; Hawkins and Ahmad, 2016). Introducing variability or “noise” in the training data or the computing units (Destexhe and Contreras, 2006; Guo et al., 2018; Wu et al., 2019) (arguably modeling their biological counterparts) can shape the properties of their representations (Faisal et al., 2008). In spatial navigation for example, this variability is useful for favoring the emergence of robust representations that resemble the spatial representations found in the medial temporal lobe (Banino et al., 2018). The robustness to environment variability, noisy sensors, and actuators of these emergent spatial representations has been proposed to be crucial in the efficiency of biological systems to navigate (Vickerstaff and Cheung, 2010).

From the AI perspective, one of the main criticisms of the current development of DL and AI in trying to understand the brain is that, recently, the main focus of such developments has been to exploit the computational power of deep architectures for technological advancement. This emphasis produced powerful classification devices that are poor at generalizing and at extracting semantic knowledge. However, such processes have been proposed to advance our understanding of intelligent behavior (Hassabis et al., 2017; Zador, 2019). Analogously, artificial autonomous navigation is an active area of AI research for engineering driverless vehicles (Lipson and Kurman, 2016). Even though one of the most popular algorithms in autonomous vehicles has a version based on certain aspects of the neuroscience of the navigation system in rodents (Milford et al., 2010; Ball et al., 2013; Xu L. et al., 2019), this particular approach has not been designed to advance what we know about the brain, suggesting a potentially unrealized opportunity for synnergy between the neuroscience of spatial navigation and AI (Dudek and Jenkin, 2002; Zafar and Mohanta, 2018).

### AI Contributions in Neuroscience Research

The contribution of AI and machine learning in neuroscience is 2-fold. At a more straightforward level, machine learning provides an analysis tool to make sense of brain activity and behavior in animal models in neuroscience. For example, DNNs have also been applied to analyze animal behavior to predict motor impairments in a mouse model of stroke. In this work, the trained network was able to predict the volume of brain tissue affected by stroke (Ryait et al., 2019). Similarly, DNNs have been used for pose estimation of animal videos (Mathis et al., 2018). In addition, and more related to spatial navigation, DNNs have also been applied to decode sensory and behavioral information such as animal position and orientation from hippocampal activity (Frey et al., 2019; Xu Z. et al., 2019). In a less direct way, AI has advanced neuroscience by providing a model of the brain. For example, DNNs have been used to reproduce brain activity in the visual system to learn about the organization of this network in primates (Walker et al., 2019) and mice (Cadena et al., 2019). Analogously and as previously mentioned, AI approaches have been used as a model of the brain to understand how spatial representations emerge and under what conditions (Banino et al., 2018; Cueva and Wei, 2018; Sorscher et al., 2019).

There are limitations and criticisms for these contributions in neuroscience. One common criticism for machine learning approaches in neuroscience is the employment of DL as a model of the brain. This criticism comes from two perspectives. One is the complexity, because by employing such models it is difficult to analyze them and to fully explain their behavior, particularly when using end-to-end approaches. However, there is progress in the development of approaches to understand the computations that complex deep ANNs carry out to produce their outputs. For example Cohen et al. (2020), propose a layer-by-layer approach in which the features represented at different layers of a deep network trained to classify images can be understood as a hierarchical architecture that gradually builds the concepts exploited by the network. A complementary approach proposed by Tishby and Zaslavsky (2015) and Alemi et al. (2019) consists of analyzing what information is passed onto the next layer, a process called “information bottleneck.” For a review of these approaches see Montavon et al. (2018). Despite this limitation, this approach might still provide controlled, reproducible experimental sand-boxes to improve our current analytical tools (that can be applied to real brain data) or to generate and test new hypotheses (Jonas and Kording, 2017). Another concern is that, in some cases, ANNs employ non-biologically plausible algorithms (Zador, 2019). For example, in some cases, popular and successful algorithms such as backpropagation are used to train such ANNs, which do not emulate the way in which the brain “learns” (Richards et al., 2019). This criticism raises the possibility that even if we can train ANNs that perform spatial navigation, this is not a guarantee that the brain might solve the task in a similar way (Burak and Fiete, 2009; Kanitscheider and Fiete, 2017). It is in these cases where a reciprocal interaction with neuroscience research can provide inspiration to propose new biologically relevant learning algorithms. For example, there are attempts to implement biologically plausible algorithms (including variants of backpropagation) to train deep ANNs (Roelfsema and Holtmaat, 2018; Sacramento et al., 2018; Pozzi et al., 2019). Therefore, imposing biologically plausible restrictions to the artificial environment, body and brain, might allow one to directly compare the solutions found by AI to their biological counterparts to determine whether these solutions might inform us about how the brain performs spatial navigation (Sinz et al., 2019).

Another criticism of the machine learning approach and in particular of using DNNs to understand brain function and ultimately, intelligent behavior, is that this approach disregards the “refinement phylogenetic” of biological organisms (Cisek, 2019). One proposal to address this is to start with the minimal cognitive functions that gave rise to navigation in simple organisms (Yamauchi and Beer, 1996; Beer and Williams, 2015). The feedback from neuroscience can provide useful insights for the advancement of such approaches (Webb and Wystrach, 2016; Graham and Philippides, 2017). Although, the neurobiological basis of spatial navigation and its relationship to learning and memory in simpler organisms like insects are not as well-understood as in rodents.

Overall, despite the great advances in the understanding of the neurobiology of the spatial navigation system in rodents, there are important open questions in neuroscience that can benefit from AI approaches. In particular, end-to-end approaches to solve navigation tasks can help in the advancement of the neuroscience of spatial navigation because the potential solutions are not restricted to the current knowledge of the experimenter. This is a very important point in the generation of new hypotheses about how the brain might solve a complex task. For example, the manipulation of spatial representations is difficult to study with current approaches in neuroscience (Kanitscheider and Fiete, 2017). This is due to the difficulty of experimental preparations and lack of tools to analyze such complex data. AI can help neuroscience research in both regards. On the one hand, a successfully trained ANN that solves a navigation task provides the opportunity to repeat and manipulate environmental conditions (e.g., sensory inputs) and parameters (e.g., network topology) to gain insights into possibly interesting avenues to follow in rodent experiments. On the other hand, it provides an opportunity to develop analytical tools to understand the complex mechanisms employed in these models and apply them to real data.

Finally, we want to clarify that the classification of the models presented here is not necessarily exhaustive, mutually exclusive or discrete. There are end-to-end models that have different levels of assumptions or use a hypothesis driven approach to different extents. Similarly, in theoretical neuroscience normative models might not be considered completely equivalent to end-to-end models in AI. However, the purpose of the classification presented in this paper was to highlight the differences of end-to-end AI approaches that can further our understanding of brain function and provide a common language that can bridge the communication gap between the neuroscience of spatial navigation and AI.

## Conclusions and Future Directions

In this paper we propose spatial navigation as a common ground where research in neuroscience and AI can converge to expand our understanding of the brain. We suggest that by understanding how the brain carries out the cognitive processes to solve a complex task such as spatial navigation, we will be in a better place to understand how intelligent behavior might arise. There are several reasons why we propose this. First, spatial navigation is a complex task that involves areas and cognitive processes in the brain that are crucial for intelligent complex behavior. Second, by using spatial navigation as a problem to be solved by artificial systems that follow biologically relevant restrictions, we can use this as a “sandbox” to improve our analytical tools. Our hope is that this paper provides a starting point for researchers of both fields to further the understanding of spatial navigation as a path to advance the study of how biological brains produce intelligent behavior.

To conclude, by building models and agents that solve spatial navigation tasks following the restrictions imposed by the interactions of the body and environment found in biological systems, we argue that we can not only learn more about the brain but also how the processes involved in complex intelligent behavior might rise. Moreover, by producing comparable solutions that can be validated against experimental results in neuroscience, we might advance the development of ANNs and overcome current limitations. Promising research avenues can be drawn from the approaches and studies presented here. For example, further investigation of learning of spatial representation at multiple scales in time and levels of abstraction, the role of memory in these processes, knowledge extraction, learning to learn, and understanding how the brain performs coordinate transformation between body-centered and map-like representations. These are crucial cognitive components of intelligence which can have a great impact in neuroscience and AI.

Despite much room for future growth, the early joint efforts between AI and neuroscience to understand the neural substrates for spatial navigation is gaining traction and is becoming an exciting and promising approach. We propose that this synergetic interaction will help to understand how cognitive processes give rise to intelligent behavior but also will contribute to the development of better tools to do so.

## Author Contributions

EB-C conceived and presented the original idea. All authors discussed the contents and contributed to the final manuscript.

## Conflict of Interest

The authors declare that the research was conducted in the absence of any commercial or financial relationships that could be construed as a potential conflict of interest.
